# Current Clinical Applications of Magnifying Endoscopy with Narrow Band Imaging in the Stomach

**DOI:** 10.1155/2012/271914

**Published:** 2012-09-16

**Authors:** Hai-Yan Li, Zhi-Zheng Ge, Mitsuhiro Fujishiro, Xiao-Bo Li

**Affiliations:** ^1^Division of Gastroenterology and Hepatology, Shanghai Jiao-Tong University School of Medicine Renji Hospital, Shanghai Institute of Digestive Disease, Key Laboratory of Gastroenterology & Hepatology, Ministry of Health (Shanghai Jiao-Tong University), 145 Middle Shandong Rd, Shanghai 200001, China; ^2^Department of Endoscopy and Endoscopic Surgery, Graduate School of Medicine, The University of Tokyo, 7-3-1 Hongo, Bunkyo-ku, Tokyo 113-8655, Japan

## Abstract

Narrow band imaging (NBI), in conjunction with magnifying endoscopy (ME), has arisen more and more attention in the area of advanced endoscopy. By enhancing the mucosal microvascular architecture and surface pattern, it is feasible to use ME-NBI to identify subtle changes associated with gastric inflammation, atrophy, intestinal metaplasia, and early gastric cancer. The new technique thus plays a valuable role in therapeutic decision-making, endoscopic treatment process, postoperative evaluation, and follow-up examination. To date, many criteria or evaluation method of ME-NBI has been proposed. This paper aims to summarize the various diagnosing classifications and the current clinical applications of ME-NBI in the stomach.

## 1. Introduction

Magnifying endoscopy (ME), for the diagnosis of gastrointestinal tract, started in the late 1960s and it has been increasingly popular since electronic videoendoscopes gradually replaced fibreoptic endoscopes [1]. With a magnified observation, endoscopists were then able to visualize the fine details of mucosal surface pattern and vascular architecture. In the 1980s, another technique in the area of endoscopy came into use, namely, the chromoendoscopy, which brought about better delineation of tumor contours and identification of mucosal pit patterns [2]. Furthermore, by narrowing the bandwidth of spectral transmittance, a narrow band imaging (NBI) system was developed in the last decade [3]. This special technique can enhance the contrast between microvessels and background mucosal surface and allow better evaluation of faint or diminutive changes. With these ongoing developments, it becomes probable to detect and differentiate gastrointestinal tumors at an early stage and modern endoscopists are moving towards the role of pathologists, that is, the optical histology. As for stomach, it is the combination of magnifying endoscopy with narrow band imaging (ME-NBI) that highlights suspicious lesions and brings better diagnostic efficacy. We herein review the recent publications and present a wide extent of the clinical applications of ME-NBI in the stomach. In general, most diagnostic criteria of NBI for gastric lesions have been proposed on the basis of previous research of ME or chromoendoscopy.

## 2. Evaluation of Gastritis, Atrophy, Intestinal Metaplasia, and Adenoma

**Figure 1:**
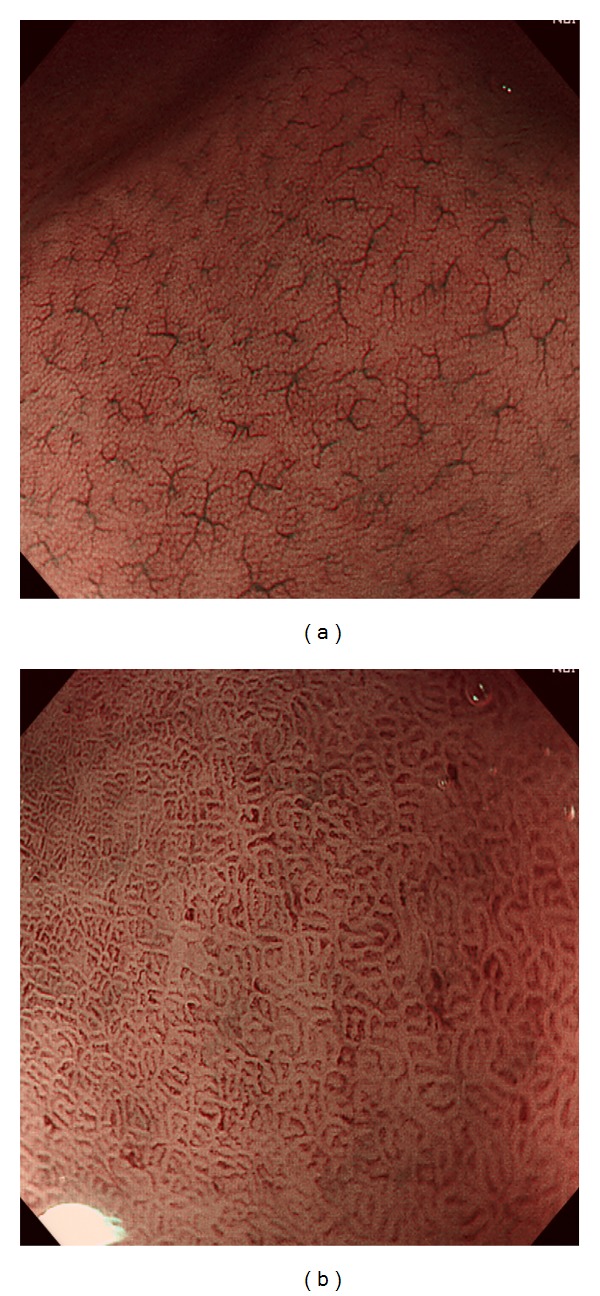
ME-NBI findings of normal gastric mucosa without HP infection. Normal gastric corpus shows small round pits, which are surrounded by honeycomb-like SECNs and interspersed with spider-like CVs (a). In normal gastric antrum, coiled or wavy SECNs surrounded by linear or reticular pits are observed and CVs are invisible (b).

Appearance of normal gastric mucosa without *Helicobacter pylori* (HP) infection has been confirmed by a series of studies. It differs depending on the location of the stomach. In gastric corpus (Figure 1(a)), ME shows small round pits which are surrounded by honeycomb-like subepithelial capillary networks (SECN) and interspersed with spider-like collecting venules (CV) [4, 5]. However, in gastric antrum (Figure 1(b)), ME demonstrates coiled or wavy SECNs which are surrounded by linear or reticular pits and CVs are rarely observed because they situate at the deeper part anatomically compared with  those in gastric corpus [5, 6].

**Figure 2:**
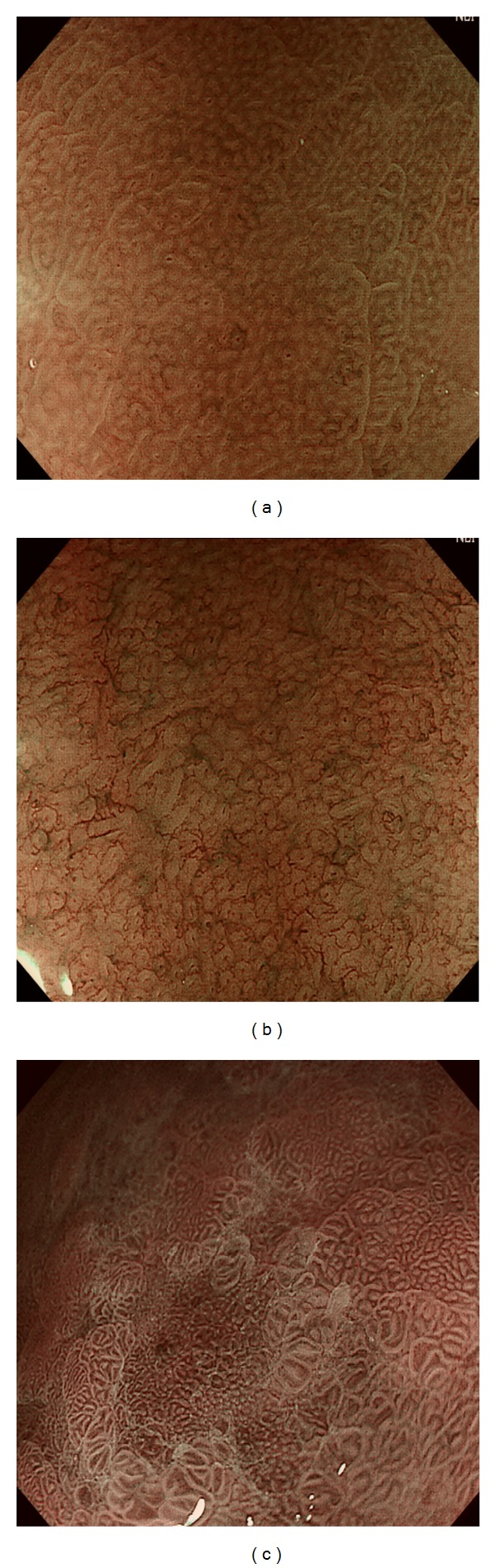
Three abnormal ME-NBI patterns in the corpus correlating with HP infection and histological severity of gastritis. (a) Slightly enlarged, round pits with unclear SECNs. (b) Obviously enlarged, oval or prolonged pits with increased density of irregular vessels. (c) Oval and villous pits in different sizes are observed and microvascular architecture demonstrates coiled or wavy vessels or a regular ultrafine network.

Therefore, nonvisualization of CVs, irrespective of pit and SECN changes, was considered suggestive of HP positive gastritis for gastric corpus by some scholars [4, 7, 8]. Moreover, Yagi et al. [7] classified the magnifying nonneoplastic mucosa of gastric corpus into four types: type Z-0 presented with CVs, regular SECNs, and gastric pits resembling pinholes; type Z-1 presented with regular or irregular SECNs but no CVs; type Z-2 presented with dilated gastric pits but neither CVs nor regular SECNs; type Z-3 presented with dilated pits surrounded by irregular redness. Type Z-0, just as the normal corpus pattern described above, correlated well with HP negative mucosa, whereas all the other types suggested HP positive gastritis. Recently, Tahara et al. conducted a similar study using ME-NBI, in which nonneoplastic mucosa of gastric corpus was also classified into four types to predict HP infection and histological severity of gastritis as well as gastric atrophy [9]. Their classification was based on the degree of irregularity of pits and microvessels, with little attention of CV changes, and consisted of a normal type and three abnormal types (Figure 2: type 1–3). Sensitivity and specificity of type 1–3 for distinguishing HP positive from HP negative mucosa were as high as 95.2 and 82.2%, respectively, and those of type 3 for diagnosing intestinal metaplasia were 73.3 and 95.6%, respectively.

With regard to gastritis of gastric antrum, a US study clarified that loss of SECN was related to HP positive gastritis [10]. They evaluated both gastric corpus and antrum with ME-NBI and reported that the sensitivity and specificity of an irregular pattern with decreased density of vessels for the diagnosis of HP infection were 75% and 88%, and those of the ridge or villous pattern for the diagnosis of intestinal metaplasia were 80% and 100%, respectively.

**Figure 3:**
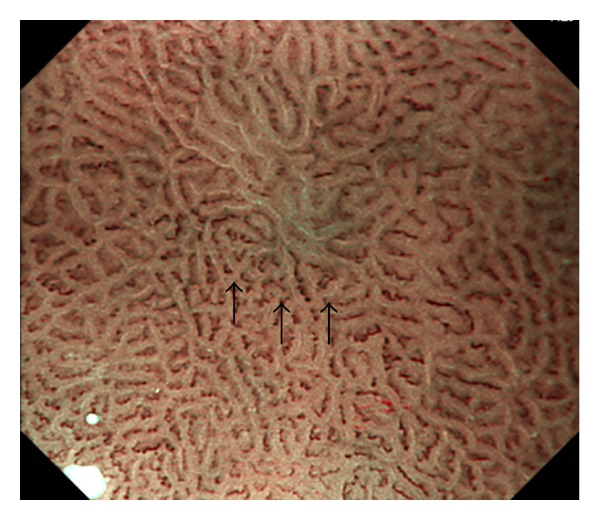
Gastritis in the antrum with atrophy and intestinal metaplasia. The mucosa (black arrow) demonstrates ridged to villous surface structure fringed by a fine blue-white line, that is, light blue crest (LBC).

In addition, Uedo et al. reported a novel finding highly suggestive of intestinal metaplasia, that is, light blue crest (LBC), defined as a fine blue-white line on the crests of the epithelial glandular structure (Figure 3) [11]. They obtained a high sensitivity of 89%, a high specificity of 93%, and a high accuracy of 91% in predicting intestinal metaplasia with ME-NBI. Compared with other diagnosing method, LBC is an obvious evidence to recognize and thus easier to acquire for nonexperienced endoscopists. Besides, in the view of NBI without magnification, LBC corresponds to the appearance of bluish-whitish patches, which may help to detect intestinal metaplasia.

Most adenomas protrude above the surface while a few have a depressed appearance together with a higher malignant potential. Tamai et al. reported that depressed adenomas showed a specific ultrafine network pattern in which the microvascular structures circled around small gland pits and formed themselves into very fine and regular network [12]. In that article, none of the tested protruding adenomas displayed these specific findings.

## 3. Differential Diagnosis between Gastric Cancer and Benign Lesions

There have been a lot of articles, as well as several ME-NBI diagnosing criteria, elucidating the efficacy of ME-NBI in distinguishing early gastric cancer (EGC) from benign lesions. In particular, it is noted that many scholars have made efforts to identify gastric cancer from biopsy-proved adenoma [13–17]. Some of the studies on differential diagnosis between gastric cancer and benign lesions using ME-NBI are summarized in Table 1.

As early as 2002, Yao et al. reported features of EGC using ME by identifying irregular microvessels and a demarcation line between the lesion and surrounding mucosa [22]. After incorporating NBI into ME examination, they paid attention to both microvascular (MV) and microsurface (MS) pattern changes and thereafter proposed the VS (vessel plus surface) classification [23]. This classification categorized MV and MS pattern separately into three types, namely, regular, irregular, and absent and set criteria for gastric cancer as “presence of an irregular MV pattern with a demarcation line” or “presence of an irregular MS pattern with a demarcation line” [23]. Based on the VS classification, several studies have performed good outcomes using ME-NBI [14, 17]. On the other hand, Ezoe et al. concentrated on the irregular MV pattern alone and also obtained a superior accuracy of ME-NBI through a prospective study using magnifying white light imaging (WLI) in the control group [20]. Moreover, a recent randomized multicenter-controlled trial has demonstrated a maximum diagnosing efficacy with sensitivity and specificity as high as 95.0% and 96.5% when irregular MV patterns on ME-NBI were in conjunction with conventional endoscopy findings [21]. In spite of these excellent results, we should note that the two studies both designated depressed or flat lesions as target lesions. With regard to elevated lesions, it has been reported that the MV pattern may sometimes be invisible due to the presence of a white opaque substance (WOS) and thus the MS pattern may be useful in diagnosing these cases [13, 17, 24].

Different from those studies that generalized all positive findings as irregular changes, Kaise et al. evaluated various ME-NBI features and put forward a specific diagnostic triad based on the most significant findings related to cancer: disappearance of fine mucosal structure (FMS), microvascular dilation, and heterogeneity [18]. Thereafter, they verified the efficacy of this diagnostic triad through additional studies on depressed or flat lesions [19, 25] and one achieved a sensitivity of 92.9% and a specificity of 94.7% compared with WLI (sensitivity, 42.9%; specificity, 61.0%) [19]. At the same time, other researchers contributed to evaluation of elevated lesions based on both MV and MS pattern and proposed their own criteria of ME-NBI [15, 16, 26]. For example, Nonaka et al. [15] classified MS pattern into clear, slightly obscured and markedly obscured, and classified MV pattern into unclear, clear, and abnormal. The diagnosing criteria, which were supposed to differentiate well-differentiated adenocarcinoma and adenoma, were then proposed: Type I (clear MS and unclear MV), Type II (clear MS and clear MV), Type III (clear MS and abnormal MV), type IV (slightly obscured MS and abnormal MV), and type V (markedly obscured MS and abnormal MV). Consequently, they showed that 79% of types I-II lesions were accurately predicted as adenoma and 93% of the types III–V lesions were accurately predicted as well-differentiated adenocarcinoma.

## 4. Differential Diagnosis between Subtypes of Early Gastric Cancer

Although high accuracies superior to conventional endoscopy have been achieved to diagnose EGC, the ability of ME-NBI to evaluate invasion depth or histological type of EGC has been less reported.

In 2010, a consensus conference on NBI diagnosis of upper gastrointestinal cancer was held by a panel of experts from Asian Pacific countries [27]. They consider that submucosal invasion may not be reflected at the mucosal surface and thus the prediction of invasive depth by minute mucosal appearance is not reliable. Consequently, they all voted to reject or disagree the proposal that ME-NBI is useful for diagnosis of tumor depth of EGC. As far, there has been no study illustrating the benefit of ME-NBI in predicting cancer invasion depth.

**Figure 4:**
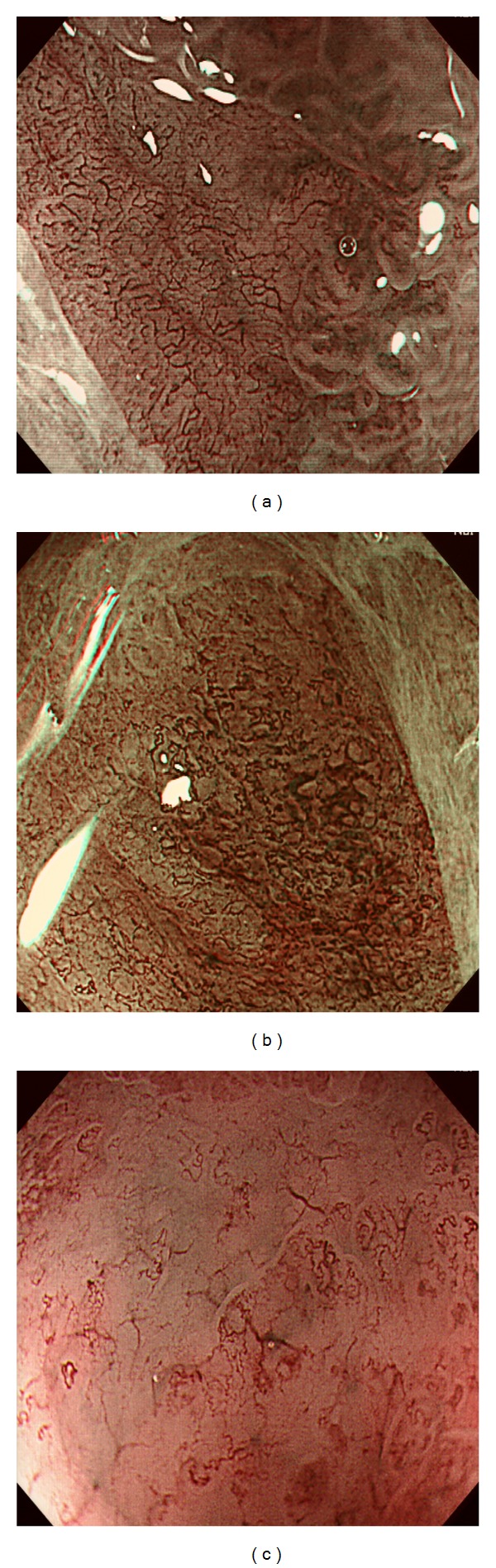
ME-NBI findings of early gastric cancer. (a) A depressed lesion showed absent surface pattern, a fine network of abnormal microvessels, and a clear demarcation line. Histology revealed high-grade adenoma with severe dysplasia (category 4.1 in the Vienna classification). (b) A well-differentiated adenocarcinoma demonstrated destructed surface pattern and dense irregular microvascular architecture: dilatation, torturous running, caliber changes, and communication of microvessels. (c) An undifferentiated adenocarcinoma showed that surface structure was almost disappeared and corkscrew-like or branched thick microvessels were sparsely distributed.

Nakayoshi et al. [28] first compared ME-NBI findings with two histological types of EGC: differentiated (D-) type and undifferentiated (UD-) type adenocarcinoma (Figure 4). Two characteristic patterns were described, one of which was called fine network pattern (FNP) with an irregular MV network and the other was corkscrew pattern (CSP) with isolated corkscrew-like vessels. Based on the analysis of 165 depressed lesions, they found that 66.1% (72/109) of the D-type adenocarcinomas exhibited an FNP and 85.7% (48/56) of the UD-type adenocarcinomas exhibited a CSP. At the same time, 23.6% (39/165) of their lesions were regarded as unclassified. Thereafter, Yokoyama et al. [29] defined a new category for the unclassified tumors, that is, intralobular loop pattern (ILL) and refined the original FNP and CSP patterns based on irregular MV and MS changes. In FNP, the abnormal MV network encircled small glandular structure, and CSP presented with absent surface structure and numerous abnormal corkscrew-like vessels. ILL-1 presented with loop-like microvessels located inside the villous surface structure and in ILL-2, the villous structure began to break apart. It turned out that all lesions having FNP and ILL-1 pattern were D-type adenocarcinomas and all but one lesion with CSP were UD-type adenocarcinomas. With regard to ILL-2 pattern, 14 of 68 lesions were UD-type and the other 54 lesions were D-type adenocarcinomas.

An interesting study assessed 120 intramucosal D-type adenocarcinomas and found that significance of the morphogenetic difference between ME-NBI patterns was related to mucin phenotype of the lesion [30]. They showed that 92.3% (24/26) of the ILL lesions were gastric or gastrointestinal phenotype and 84.6% (22/26) of the FNP lesions were intestinal phenotype. As for lesions showing combined or unclear findings of ILL and FNP, 73.5% (50/68) were gastrointestinal phenotype.

For UD-type intramucosal cancer, the cancer development progresses from intermediate or deep layer to superficial layer and finally into the whole layer. Some scholars found that different ME-NBI patterns, such as S-type with preserved but irregular surface structure and V-type with absent surface structure and irregular microvascular architecture, were correlated with the development period of UD-type cancer [31]. In their study, all lesions (24/24, 100%) with S-type corresponded to intramucosal UD-type cancer located in nonwhole layer. The percentages of V-type lesions (including lesions with both types) corresponding to nonwhole-layer intramucosal cancer whole-layer intramucosal cancer, and submucosal invasive cancer, were 27.8% (15/54), 50.0% (27/54), and 22.2% (12/54), respectively.

In either CSP or V-type lesions, the appearance shows nonstructural which means the MS pattern is absent. Correspondingly, disappearance of fine mucosal structure has also been mentioned in the diagnostic triad proposed by Kaise et al. However, the latter was considered as indicative for cancer whereas the former ones, in a deeper perspective, were considered as indicative for UD-type cancer. According to colorectal Kudo's classification [32], a nonstructural structure means deep submucosal invasion. In terms of EGC, a prospective study enrolling 50 EGCs and 11 adenomas revealed the clinical meaning of the nonstructural pattern with ME [33]. They found that all adenomas and 93.5% (29/31) mucosal-differentiated cancers did not show the nonstructural pattern, whereas nine of the 11 submucosal cancers showed the nonstructural pattern. The disappearance of MS pattern tended to be identified in UD-type cancer or cancer with submucosal invasion.

## 5. ME-NBI for Evaluation of Tumor Margin

The wide use of endoscopic resection for EGC directly accelerates the need for accurate evaluation of the lesion, including accurate assessment of horizontal extent of the cancer preoperatively. With the ability of highlighting subtle mucosal changes, chromoendoscopy, especially in conjunction with magnifying endoscopy, has consistently been a popular method for preoperative evaluation of the lateral spread. However, Nagahama et al. [34] reported that it was difficult to determine the margins in about 20% of EGCs by using magnifying chromoendoscopy. They also found that ME-NBI was able to delineate the entire margins in 72.6% (45/62) of the lesions that had shown unclear margins using chromoendoscopy. Additionally, a randomized study compared the usefulness for determining the tumor margin between ME-NBI and chromoendoscopy with indigo carmine dying [35]. They found that the success rate of margin delineation of the ME-NBI group was significantly higher than that of the chromoendoscopy group (97.4% versus 77.8%, *P* = 0.009).

In conventional endoscopy, chromoendoscopy, or even nonmagnifying NBI observation, a demarcation line can be identified with reference to depressions, elevations, or color changes of the lesions compared with surrounding mucosa. However, by using ME-NBI, we can further evaluate the demarcation line and make additional judge. According to the VS classification [23], the real cancer-specific margin lies between the interior which shows an irregular MV architecture or irregular MS pattern and the surrounding mucosa with regular MV and MS pattern.

In spite of the superior accuracy reported in several studies [23, 34–36], the margins remain difficult to determine for UD-type cancers because they often develop laterally within the lamina propria sparsely or diffusely before exposure on the mucosal surface. As a result, no clear demarcation line can be detected in ME-NBI. So prior to endoscopic treatment, multiple biopsies of the background mucosa are necessary to confirm the real negative circumstances. In contrast, Okada et al. [31] proved that UD-type cancer was still detectable when confined to the nonwhole layer. They clarified that these cases showed the S-type with comparatively larger microsurface structures and wider spaces between crypts. Applying the S-type and V-type criteria (mentioned above), they successfully used ME-NBI to predict the lateral extent of cancer in all 18 consecutive UD-type EGCs resected by ESD.

## 6. Application of ME-NBI in Gastric Mucosa-Associated Lymphoma

Compared with the epithelial tumors, only a few studies have been reported on the benefit of ME-NBI in diagnosing nonepithelial tumors. Gastric mucosa-associated lymphoid tissue (MALT) lymphoma is an indolent type of lymphoma, characterized with distinct clinicopathological features and complete remission (CR) is possible after HP eradication or radiation therapy. So it is necessary to differentiate it from EGC, such as UD-type cancer. According to recent studies [37–39], characteristic ME-NBI findings of MALT lymphoma are nonstructural pattern (complete or almost complete disappearance of gastric pits), abnormal vessels (irregular in size and formation, not seen in normal mucosa), and swelling crypt epithelium. Moreover, the large vessels like a tree trunk with long bare branches are more unique and specific. A retrospective study [39] has clarified the utility of these findings in diagnosing MALT lymphoma and in evaluating remission: nonstructural pattern was most reliable in diagnosing MALT lymphoma with a high sensitivity (94.6%) and specificity (100%); disappearance of abnormal vessels was most reliable in predicting remission with a sensitivity (85.7%) and specificity (85.7%). However, the background population was set as MALT lymphoma patients and no other patients were enrolled. In other studies, mantle cell lymphoma was also reported to have such features [40]. Moreover, UD-type cancer sometimes shows nonstructural pattern and markedly abnormal vessels which are similar to these findings [29, 31]. So it remains difficult to distinguish MALT lymphoma from other malignant entities using ME-NBI only. But the utility of ME-NBI in directing target biopsy and evaluating complete remission of MALT lymphoma is very meaningful in clinical practice.

## 7. ME-NBI for Treatment, Evaluation and Followup

ME-NBI has been used as a tool to predict the results of HP eradication [41], remission condition of MALT lymphoma [38], identification of recurrent EGC after previous endoscopic resection [42], and so on.

Okubo et al. [41] investigated gastric mucosal patterns of the same site before and after HP eradication therapy and found out that change in ME-NBI pattern had a sensitivity of 83.3% and a specificity of 100% in predicting outcomes of HP eradication therapy. In their study, 20 of 24 patients who were successfully treated showed remarkable changes, that is, enlarged or elongated pits improved to small oval or pinhole-like round pits, and the density of irregular vessels decreased. It has also been demonstrated that change of ME-NBI pattern seldom happened in patients with severe gastric atrophy and intestinal metaplasia, even if HP had been eradicated.

Ono et al. reported consecutively the findings of ME and ME-NBI for patients with MALT lymphoma before and after treatment [38, 39]. As mentioned above, MALT lymphoma showed disappearance of gastric pits and appearance of abnormal vessels. After successful treatment, gastric pits and subepithelial capillary network recovered and abnormal vessels disappeared. With magnifying or ME-NBI observation, endoscopists may either estimate responders and nonresponders for treatment without biopsy confirmation or obtain targeted biopsies from areas likely to contain residual disease. In addition, ME-NBI helps to determine the situation of HP infection and thus aids in selecting treatment strategy for MALT lymphoma.

**Figure 5:**
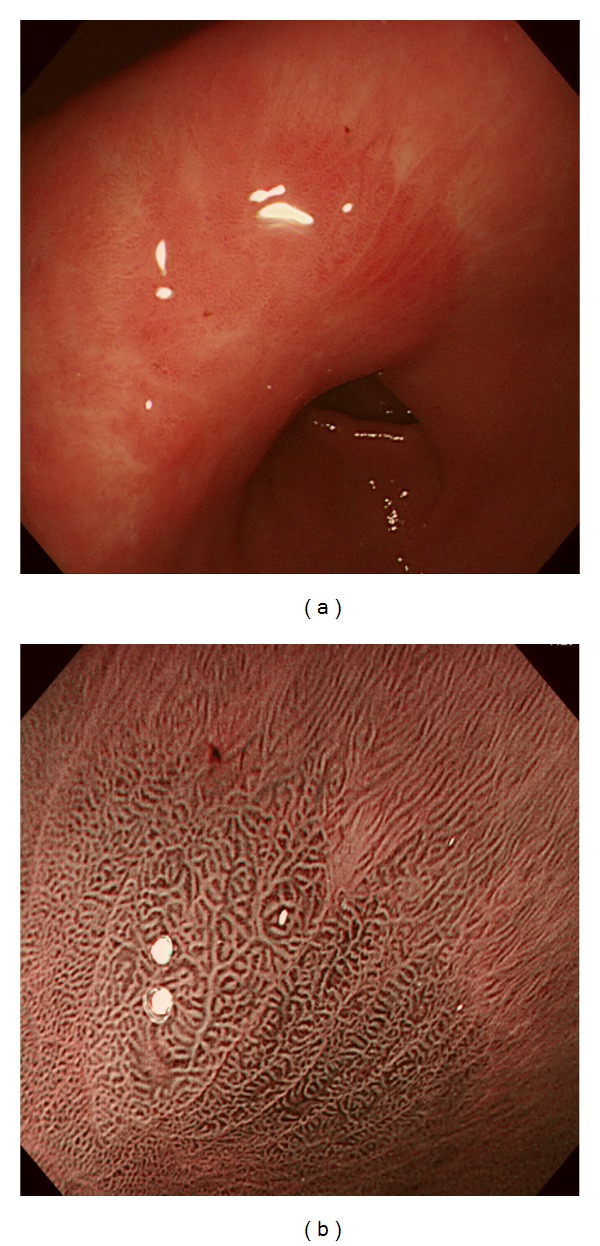
Evaluation of a post-ESD scar six months later. Conventional endoscopy showed a circle scar and regenerated glandular epithelium (a). ME-NBI observation of the scar showed regular regenerated surface structure and regular SECNs encased in the mucosal crest (b).

Endoscopic resection (ER) is popular for management of gastric lesions and both short-term and long-term follow-up outcomes are vital. However, less has been reported regarding the correlation of endoscopic findings and pathological findings in post-ER scars, as well as a detailed description of ME-NBI findings in the altered mucosa (Figure 5). In 2009, a prospective short-term follow-up study investigated the relationship between magnifying findings of post-ER scars and the pathological diagnosis [43]. They found that the presence of nodularity on conventional endoscopy suggested tumor lesions with a sensitivity of 88.9% and a specificity of 62.5%. On the other hand, a destroyed pit pattern on magnifying endoscopy has a sensitivity and specificity both reaching 100% in predicting tumor lesions. Besides, Kosaka et al. [42] reported the efficacy of several modalities in identifying and demarcating residual or local recurrent gastric neoplasm after ER, including conventional ME, enhanced-ME with acetic acid instillation (EME), ME-NBI, and NBI-EME. It turned out that a combination of NBI or acetic acid instillation was very effective in ME for post-ER followup.

## 8. Conclusion

ME-NBI is a feasible and efficient endoscopic technology that can improve diagnostic accuracy for precancerous lesions and early cancers in the stomach. Furthermore, this new technique enables us to make better evaluation of gastric cancers both preoperatively and postoperatively, such as tumor contour, histology, and possible invasion depth, as well as follow-up results. Despite that a lot of meaningful clinical studies have been published with good results, internal reliability or external validity of ME-NBI has seldom been evaluated. Some studies were conducted at a single center or performed by a single endoscopist and no study enrolled the whole spectrum of gastric lesions. Moreover, results of a few studies seem to contradict with those of others. A large-scale, multicenter, prospective, and randomized trial is necessary to confirm the efficacy of ME-NBI and standardize the diagnostic criteria.

## Figures and Tables

**Table 1:** Results of selected studies of differential diagnosis between gastric cancer and benign lesions using ME-NBI.

Author	Year	Morphology	Study methods	ME-NBI criteria	Patients (*n*)	Results
Kaise et al. [18]	2009	Depressed	Blinded review of images	Diagnostic triad: disappearance of MS, MV dilation, and MV heterogeneity	100	Sensitivity and specificity: ME-NBI with the triad (69%, 85%), WLE (71%, 65%), and ME-NBI general (72%, 80%)
Kato et al. [19]	2010	Depressed, or flat	Prospective, comparative	Diagnostic triad: disappearance of MS, MV dilation, and MV heterogeneity	111	ME-NBI > WLE; ME-NBI: sensitivity (92.9%), specificity (94.7%)
Ezoe et al. [20]	2010	Depressed, or flat	Prospective comparative, real-time diagnosis	Irregular MV with a demarcation line	53	ME-NBI > ME-WLI; ME-NBI: sensitivity (70%), specificity (89%)
Ezoe et al. [21]	2011	Depressed	Multicenter randomized and controlled, real-time diagnosis	Irregular MV with a demarcation line	353*	ME-NBI + C-WLI > ME-NBI > C-WLI; ME-NBI + C-WLI: sensitivity (95.0%), specificity (96.8%), and accuracy (96.6%)
Nonaka et al. [15]	2011	Elevated	Prospective, multicenter	Type I: clear MS, unclear MV; type II: clear MS, clear MV; type III: clear MS, abnormal MV; type IV: slightly obscured MS, abnormal MV; type V: markedly obscured MS, abnormal MV	93	Types I-II: 79% as adenoma; type III–V: 93% as well-differentiated adenocarcinoma
Miwa et al. [17]	2012	Depressed, and elevated	Retrospective, comparative; blinded review of images	Irregular MV and/or irregular MS with a demarcation line	135	Elevated lesions: ME-NBI > WLI (sensitivity, 82.4 versus 70.6%; specificity, 97.3 versus 54.7%); depressed lesions: sensitivity, ME-NBI > WLI (95.5 versus 68.2%); specificity, ME-NBI = WLI (100 versus 100%, *P* > 0.99)

ME-NBI: magnifying endoscopy with narrow band imaging; WLI: white light imaging; C-WLI: conventional WLI; MV: microvascular; MS: microsurface.

*Patients for final analysis.
